# Nearest Consensus Clustering Classification to Identify Subclasses and Predict Disease

**DOI:** 10.1007/s41666-018-0029-6

**Published:** 2018-07-30

**Authors:** Awad A. Alyousef, Svetlana Nihtyanova, Chris Denton, Pietro Bosoni, Riccardo Bellazzi, Allan Tucker

**Affiliations:** 1 Department Computer Science, Brunel University London, Uxbridge, UK; 2 UCL Royal Free Hospital, London, UK; 3 University of Pavia, Pavia, Italy

**Keywords:** Consensus clustering, Disease subgroup discovery, Classification

## Abstract

Disease subtyping, which helps to develop personalized treatments, remains a challenge in data analysis because of the many different ways to group patients based upon their data. However, if we can identify subclasses of disease, then it will help to develop better models that are more specific to individuals and should therefore improve prediction and understanding of the underlying characteristics of the disease in question. This paper proposes a new algorithm that integrates consensus clustering methods with classification in order to overcome issues with sample bias. The new algorithm combines K-means with consensus clustering in order build cohort-specific decision trees that improve classification as well as aid the understanding of the underlying differences of the discovered groups. The methods are tested on a real-world freely available breast cancer dataset and data from a London hospital on systemic sclerosis, a rare potentially fatal condition. Results show that “nearest consensus clustering classification” improves the accuracy and the prediction significantly when this algorithm has been compared with competitive similar methods.

**Electronic supplementary material:**

The online version of this article (10.1007/s41666-018-0029-6) contains supplementary material, which is available to authorized users.

## Introduction

Disease subtyping helps to develop personalized treatments that better fit individual patients. It does, however, remain a challenge in data analysis because of the many different approaches to clustering patients based on their data. Nevertheless, if we can identify subclasses of disease, then it will assist the development of better models that are more specific to individual groups of patients and should therefore improve prediction and understanding of the underlying characteristics of the disease in question. Cluster techniques have an effective track record in this field. Clustering methods that divide (sometimes thousands of) patients into subgroups of manageable portions can offer many advantages in medicine [1]. However, the quality of traditional algorithms such as K-means, DB-scan, and Fuzzy C-means can be both biased and variable (due to limited samples, inherent model bias and noise). For this reason, consensus clustering approaches have been developed [2]. These approaches have typically dealt with model bias and variability but not sample variance which this paper will explore through resampling approaches.

Discovering subtypes have become increasingly important as more data becomes available. Wu et al. identify clear cell renal cell carcinoma (ccRCC) as one of the most important subtypes of renal cell carcinoma. This paper highlights the importance of molecular typing for individuals for the personalized care of cancer as well as improving overall accuracy. Unsupervised consensus clustering has been used in order to discover a new subpopulation of ccRCC. An unsupervised consensus clustering approach has enabled the identification of three distinct subtypes based on hierarchical clustering. This is highly important because of the ability to identify stable categories with gene expression patterns. Also, the clusters show clinical meaning which may be informative of tumor behavior and prognosis [3]. Zhu et al. proposed a novel subspace clustering guided unsupervised feature selection (SCUFS) model. This algorithm learns by representation based subspace clustering. This algorithm learns the data distribution in that it uncovers the underlying multi-subspace structure of the data. The results show that subspace clustering guided unsupervised feature selection model outperform other approaches [4].

Choosing the right clustering method is not an easy task as different methods can return different results. Combining the results of several methods can lead to better groupings. Moreover, bootstrap techniques can be used to resample datasets in order to build more confidence in clusters [5]. Consensus clustering which explores the consensus over different clustering algorithms can increase overall confidence compared to each individual input cluster method [6]. An even higher confidence can be given to “robust clusters” which enforces maximum agreement across input clustering methods [6]. Swift et al. used robust and consensus clustering in order to improve the confidence in discovered clusters [7]. For a good review of consensus clustering methods, please see [8]. Weighted-kappa can be used to evaluate the consistency of clustering results. This statistical metric measures the interagreement among decisions made by two or more observers. It can therefore be used to compare different allocations of data to clusters and generates a score that ranges between − 1 and + 1 from poor agreement strength to very good agreement strength [9].

Having identified subclasses of patients, supervised learning can be used in disease prediction. Decision trees and Bayesian classifiers perform well [10] and have the added advantages of being transparent in how they model the data (unlike many black box approaches). Tucker et al. incorporated a model that combined unsupervised learning to identify subclasses with supervised learning to predict health outcomes for patients [11]. The results showed that it both improved the prediction and enabled better understanding by clinicians [11]. We expand on this by exploring how consensus methods can be used to identify individual models for each discovered subgroup, which aids understanding as well as improving prediction.

In this article, we have analyzed patients affected by systemic sclerosis (SSc). The aim of this article is to combine unsupervised learning that identifies potential subclasses and supervised learning that helps to predict health outcomes based upon these subclasses. We have designed a novel algorithm that has performed better than supervised learning alone by incorporating unsupervised learning (K-means clustering). We have named this algorithm *nearest consensus clustering*. In the next section, the disease will be explained followed by the data and methods in Sect. 3, including the new algorithm. We will then describe the set of experiments undertaken, and in Sect. 4, the results are documented before conclusions are drawn. In particular, thanks to a partnership between the Computer Science Department at Brunel University London and the Centre for Rheumatology and Connective Tissue Diseases (CTDs) at the Royal Free London Hospital, it was possible to work on a dataset of more than 600 systemic sclerosis subjects with the disease onset between January 1995 and December 2003, followed for up to 15 years.

## Systemic Sclerosis

In order to allow for a better understanding of our paper, it is important to state briefly the definition of systemic sclerosis (SSc) illness which is the main clinical problem in our research. Systemic sclerosis is an uncommon connective tissue disorder with multisystem involvements and a chronic and often progressive course [12]. The comparison and interpretation epidemiological studies have become quite difficult not only because of the rarity and clinical heterogeneity of SSc but also the lack of universally used classification and diagnosis criteria [13]. The understanding of the above disease and its stages has been improved. However, the causes of SSc are still unclear. There are three key pathophysiologic processes that account for its occurrence: vasculopathy of small vessels, immune response leading to production of autoantibodies, and vascular fibrosis in multiple organs [14].

The research community has validated a few clinical outcome measurements for specific SSc manifestations. The thickened skin is the main characteristic for SS illness, so there are skin thickness assessments in 17 different anatomic surfaces. The total skin score can range from 0 (no thickening) to 51 (severe thickening). The patterns of skin involvement are the most widely accepted clinical method of dividing SSc into groups [15]. The clinical test is the main criteria to diagnose the SSc. Skin induration, with a characteristic symmetric distribution patterns, institutes the diagnosis with high degree of confidence. Thickness biopsy can make certain of the disease occurrence [12]. Also, ACA (anti-centromere antibodies), ATA (anti-topoisomerase I antibodies), and ARA (anti-RNA polymerase III antibodies) are highly specific to predict SSc [16].

In SSc, each organ can be affected but some are clearly more affected than others. The gastrointestinal tract involvement is the most region that might be affected by SSc. Up to 90% of patients could have complications in any site of gastrointestinal tract [17]. Also, pulmonary fibrosis complications can be found in about 75% patients and it could affect small areas of the lung [18]. Pulmonary arterial hypertension is another serious complication of SSc that develops usually later in the disease and nonspecific symptoms [19]. The other complication is scleroderma renal crisis that is rare but very severe and life-threatening complication, one of the few medical emergencies in rheumatology [20].

## Data and Methods

### Data

Systemic sclerosis is an uncommon connective tissue disorder with multisystem involvements and a chronic and often progressive course [12]. The diagnosis of systemic sclerosis is made on the clinical grounds, and it is generally plain in patients with established disease. In fact, the presence of skin induration, with a characteristic symmetric distribution pattern associated with typical internal organ manifestations, establishes the diagnosis with a high degree of confidence, while a full-thickness biopsy of the skin is sometimes required to make certain of its occurrence [12]. Digital pitting scars and radiologic evidences of pulmonary fibrosis are useful to perform a diagnosis as well as the Raynaud’s phenomenon, although for this sign, a nailfold capillaroscopy can be requested. This procedure is a non-invasive, low-cost, and reproducible imaging method allowing the evaluation of structural changes in peripheral microcirculation, which is mainly used in the differentiation between primary and secondary Raynaud’s phenomenon [21].

The 677 patients in our data have the following distinct features:

#### General and Subset Data

- Subset: char indicating the systemic sclerosis subcategory, with only two possible options. Patients without skin thickening in areas proximal to elbows and knees were grouped into the limited cutaneous subset (L), whereas patients with skin thickening that acted both areas distal and proximal to elbows and knees were grouped into the diuse cutaneous subset (D); it is marked as “2” when it is with skin thickening and “1” when it is without skin thickening. Its values take only “1” or “2” (binary).
- Gender: char indicating the sex of patient, “m” for males and “f” for females; in the dataset, it takes “1” or “2,” where “1” refers to M and “2” refers to F.
- Age: number indicating the years of patient at disease onset (integer values).

#### Blood Tests Results

- abs: string indicating the detected autoantibodies. Next to it, there is a list of 16 columns, each one labeled as a specific autoantibody acronym and filled with a binary value to indicate its absence or presence; e.g., “0” is absent and “1” is present (binary values).
- Hb: value indicating the measure of hemoglobin; it is expressed in grams per deciliter. Normal range for men 13.5 to 17.5 g per deciliter and normal range for women 12.0 to 15.5 g per deciliter.
- Cr: value indicating the measure of creatinine in that test. It tells your doctor your stage of kidney disease. It can be calculated by serum creatinine level, age, sex, and race. Baseline for Cr is between 60 and 90 ml/min/1.73 m^2^.

#### Lung Function Test Results

- FVC: value indicating the measure of forced vital capacity in that test; it is expressed in liters.
- DLCO: value indicating the measure of diffusing capacity for carbon monoxide. It is expressed in liters.
- T2RIP: number of months between disease onset and death.
- T2PF: number of months between disease onset and pulmonary fibrosis.
- T2PAH: number of months between disease onset and pulmonary arterial hypertension.

#### Anti-Body Information

The following antibodies are marked in the dataset as binary values “1” or “0”:

- ACA is the most frequently discovered, and it is associated with the limited cutaneous subset of SSc, although a small proportion of ACA-positive patients can develop a diffuse cutaneous SSc [22].
- ATA also known as anti-Scl-70, is associated with a higher prevalence of arthritis, tendon friction rubs, severe pulmonary fibrosis, cardiac involvement, and scleroderma renal crisis [22].
- ARA are strongly associated with the diffuse cutaneous subset and correlated with severity of skin involvement [23].

Every organ can be clinically affected due to systemic sclerosis, so we are particularly interested to go explore different organ complications in this study since we want to predict the occurrence of these estimating if they might happen before or after a specific temporal threshold to better intervene. For instance, pulmonary arterial hypertension (PAH) is serious complication of SSc; it can affect both subsets in similar proportions, and it develops usually later in the disease as a debilitating and progressive disorder characterized by a blood pressure increase in arteries of the lungs. It is defined by right heart catheterization as a mean pulmonary arterial pressure not less than 25 mmHg with a pulmonary capillary wedge pressure not greater than 15 mmHg. The natural history of SSc-associated PAH is variable, but in many patients, it follows a downhill course with the development of right heart failure and death. It usually presents with nonspecific symptoms of exertional dyspnea, fatigue, angina, and exertional near-syncope. With the disease progression, symptoms and signs of right ventricular failure appear [12].

SSc shows heterogeneous clinical manifestations with a wide variability in presentation, severity, and outcome: some patients reveal fast and fatal progression, whereas others have a benign course [24]. Then, considering the disease susceptibility, there are three principal factors: age, gender, and ethnicity. Similar to other autoimmune connective tissue diseases, women are almost four times more likely than men to develop SSc; this strong female predominance is most pronounced in the childbearing years and declines after menopause [24].

We explore SSc data, provided by Royal Free London Hospital for 677 patients where we want to predict time to death and time to PAH—a common comorbidity in SSc. The aim of our proposed algorithm is to cluster the patients within three groups and to predict time to develop PAH and to predict time to death for each group. The patients have been selected as follows:

- Select all patients from the original dataset who died within the first 5 years and all patients who still alive over 5 years. The predicted class will have two values “1” representing patients who could die before 5 years and “2” representing patients who could die after 5 years. The novel algorithm was applied on this resulted dataset in order to predict time to death.
- Select all patients from the original dataset who develop PAH within the first 5 years and all patients who still not develop PAH over 5 years. The predicted class will have two values “1” which means the patient could develop PAH before 5 years and “2” the patient could develop PAH after 5 years. Also, the novel algorithm was applied on this resulted dataset in order to predict time to develop PAH.

We also explore our approach on a freely available breast cancer data provided by the UCI machine learning repository. It consists of 10 attributes and 699 patients, where we want to predict whether a tumor is benign or malignant.

## Consensus Clustering

Consensus clustering involves combining multiple cluster results. It takes a number of different clustering methods as inputs in order to find a single consensus clustering that is a better fit than each individual clustering method. Consensus clustering is needed because it represents a way of reconciling clustering information which arises from different experimental sources or from multiple runs of the same nondeterministic algorithm [25]. It is also a method of finding clusters that are more stable and less sensitive to starting values based on a membership principle. It considers multiple input clusterings where items that have been clustered repeatedly together in the inputs will be more likely to appear in the consensus clustering. For example, consensus clustering can use different clusterings as inputs that have been generated with different clustering methods or starting parameters [26] in order to remove bias. Alternatively, input clusterings can be generated by resampling the original dataset in order to generate a more stable consensus clustering by removing sampling bias.

The first task to build consensus clustering involves the construction of an *n x n* “agreement matrix” based on input clustering results. This matrix contains cells that represent the number of agreements among the input clustering methods used for clustering together each pair of objects, represented by the indexing row and column. This matrix is then employed to group objects based on their cluster agreement by rewarding clusters with high agreement between members and penalizing clusters with low agreement [9].

**Fig. 1:**
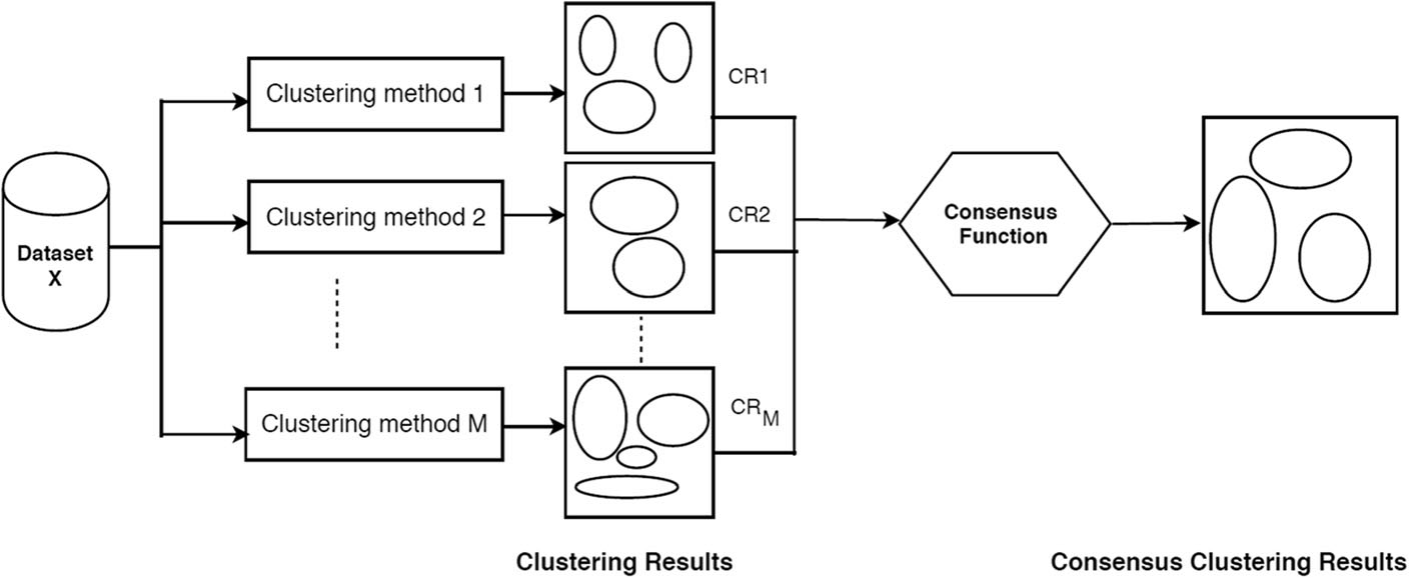
Consensus clustering algorithm (schematic)

The input methods used to generate the agreement matrix can be from the results of different clustering methods as was explored in [7]. However, here, we are concerned with sampling bias so we use different clustering results from K-means applied to repeated resampling of the data. Consensus clusters are built that reward variables if they have high cluster agreement and penalizes variables if they have low agreement. Figure 1 shows a general schematic of how consensus clustering works [27].

## Nearest Consensus Clustering Algorithm

**Fig. 2:**
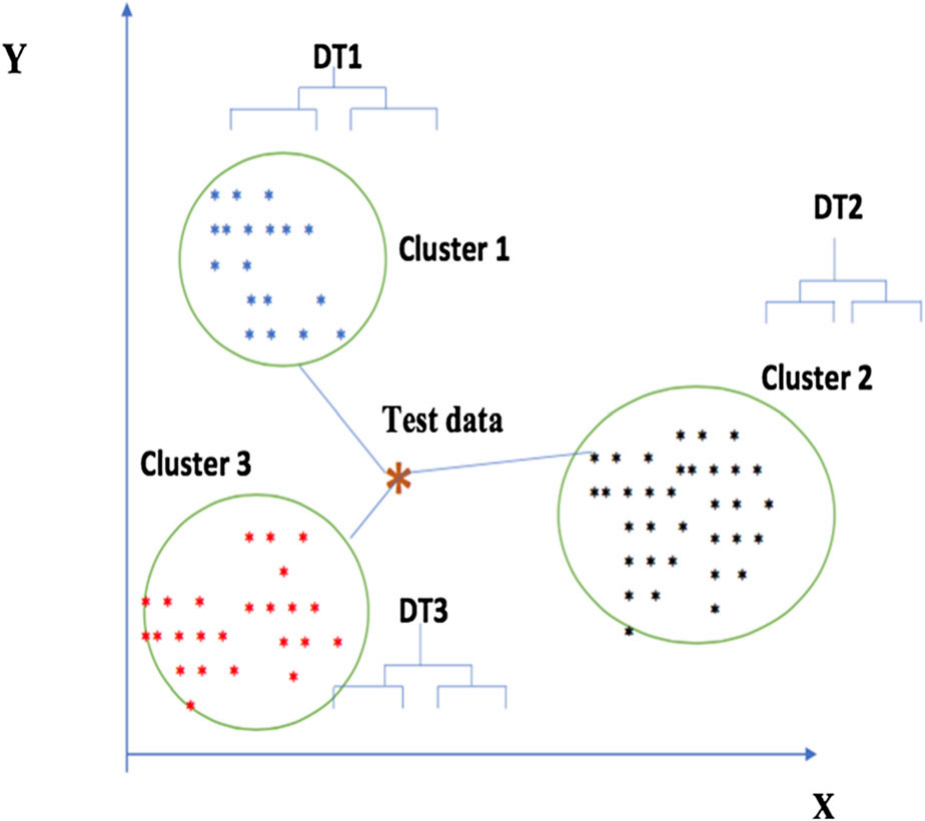
Nearest consensus clustering classification: training and testing data (schematic figure)

Our proposed method attempts to deal with the natural variation in many clustering methods as well as sample variance by using the consensus approach in combination with C4.5 decision tree classifiers. C4.5 is a decision tree method used for classification that is transparent in that it generates a tree structure that can be interpreted. The tree is inferred based on the information gain ratio measure [28]. Data is split into a training and testing set. The training data undergoes resampling to build a set of consensus clusters. A separate tree is then inferred from each of these consensus clusters. Next, each test data point is scored based upon the distance to each discovered consensus cluster using a single linkage approach with Euclidean distance. This is used to assign the appropriate decision tree to be used to classify the data point. We explore a number of distance metrics within this, e.g., single linkage, further linkage, and average linkage. Figure 2 is a general schematic figure that explains the proposed nearest consensus clustering algorithm. In this example, the training data has been divided into three clusters, using consensus clustering of multiple K-means with resampled data. The decision tree (DT) is then constructed from each consensus cluster. When classifying test data, our algorithm aligns the test data (denoted by an “x”) to the nearest consensus cluster (here cluster 3) by using the single linkage measure (nearest neighbor). The associated decision tree is then used to classify the test data point (here DT3).

The following pseudocode explains the steps that are used orderly to build the new algorithm.

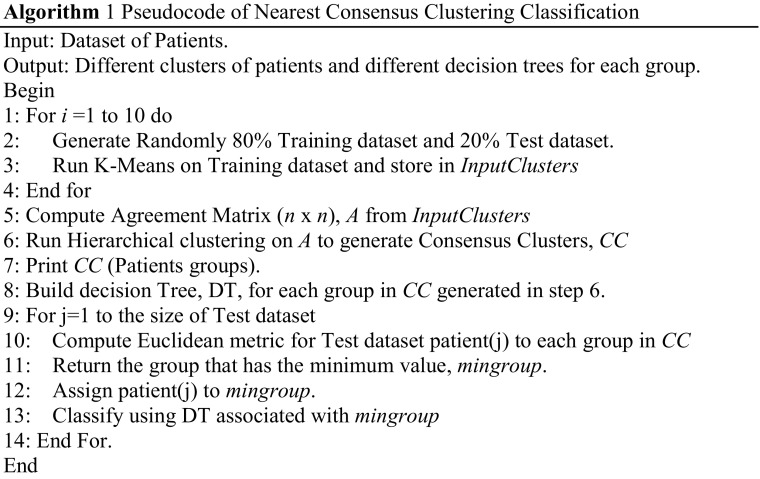



## Experiments

In this paper, we compare nearest consensus clustering classification to results with standard K-means clustering of patients, the C4.5 Decision Tree (with no clustering of patients), and nearest K-means (without consensus clustering).

In detail, we apply out nearest consensus clustering by running K-means on the training data for ten repeated resampled datasets in order to produce an agreement matrix. This aims to capture the sampling bias. K-means clustering is then applied to the agreement matrix to create the consensus clusters. Cross-validation which is an evaluation technique used to assess the predictive capabilities of a specific model on unseen examples is used to determine the accuracy. It is performed by partitioning the original data into a training set to learn the model, and a test set to evaluate it, then crossing-over both the training and validation sets in multiple iterations so that each data point is used for validation [27]. The current datasets in this were randomly resampled into training dataset 80 and 20% for ten times.

In the first set of results, we explore three methods:

i) Using simple K-means alone to identify clusters (with no resampling/consensus) for building each decision tree—we call this “nearest K-means.”
ii) A standard decision tree with no clustering at all.
iii) The full nearest consensus clustering algorithm described above.

We explore these based upon the resulting decision trees, the cluster membership, the predictive accuracy, and Kaplan-Meier curves for

A) The SSc for predicting time to pulmonary hypertension
B) The SSc for predicting time to death
C) The Breast Cancer data for predicting tumor type

We then perform the following analyses:

D) We perform a full sensitivity analysis of these methods.
E) We explore the impact of changing the number of clusters, K, on the accuracies.
F) We compare our proposed approach with other similar combinations of clustering/classifiers. In particular, we have explored hierarchical clustering and PAM and hierarchical clustering as opposed to K-means, and support vector machines (SVMs) instead of decision trees. SVM is the often considered the most consistently accurate classifier. The disadvantage of this algorithm is the complexity of determining the number of support vectors. It works by transforming data and conducting a simple scaling so that the classes are linearly separable [29]. PAM clustering is a similar method to K-means in that it splits the dataset into K groups but here medoids (rather than centroids) must be represented by a data point. These data points correspond to the most centrally located point in each cluster [30]. Hierarchical clustering is another clustering method that partitions the dataset into groups using a dendrogram tree structure [31].
G) Finally, we explore a small follow up piece of data analysis on the discovered groups within the clinical context.

## Results

### Systemic Sclerosis: Time to Develop Pulmonary Arterial Hypertension

**Fig. 3:**
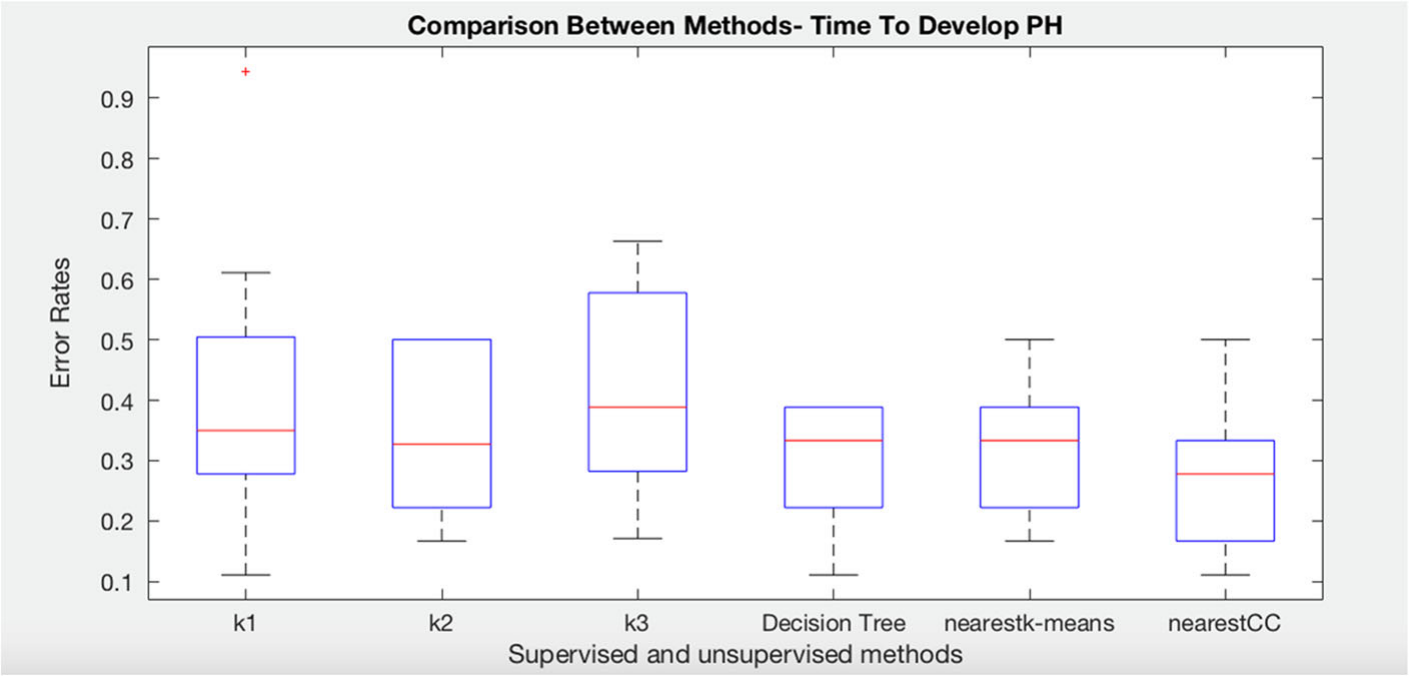
Comparison of K-means, decision tree, nearest K-means, and nearest CC for time to develop pulmonary arterial hypertension class in systemic sclerosis dataset

We have run C4.5 Decision Tree (without clustering), nearest K-means (without consensus), and nearest consensus cluster classification to the systemic sclerosis data in order to predict time to develop pulmonary arterial hypertension. The following plot (Fig. 3) shows the results of these experiments as well as the result of each individual cluster model on all of the test data (K1, K2, and K3). Notice first that each individual cluster model classifies the test data worse than ones that attempt to model all clusters. In addition, the standard decision tree and nearest K-means produce a better and less variable set of errors. Nearest consensus cluster classification performs better than all other algorithms with lower error and reduced variance (significantly better than nearest K-means with *t* test, *p* = 0.040), indicating that sampling bias is an issue that need to be addressed when identifying patient subgroups.

**Fig. 4:**
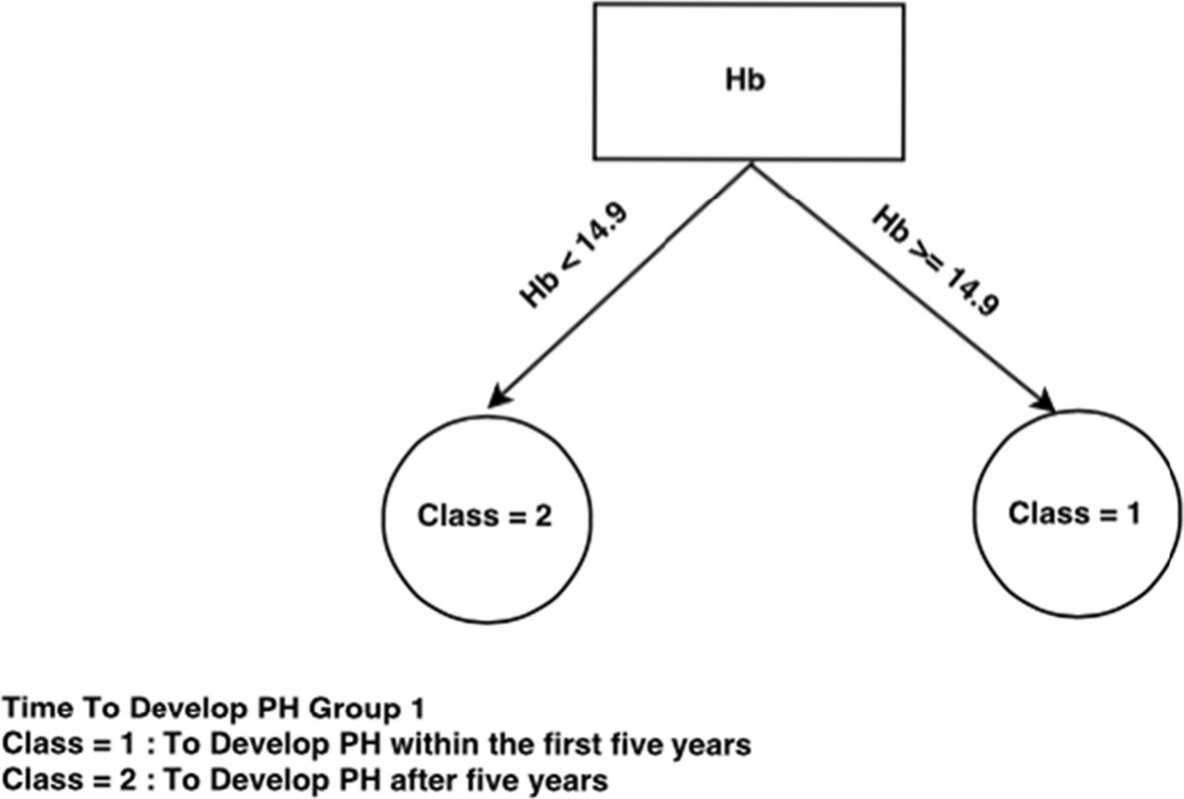
Consensus clustering decision tree for group 1 in SS dataset and time to develop pulmonary arterial hypertension class

**Fig. 5:**
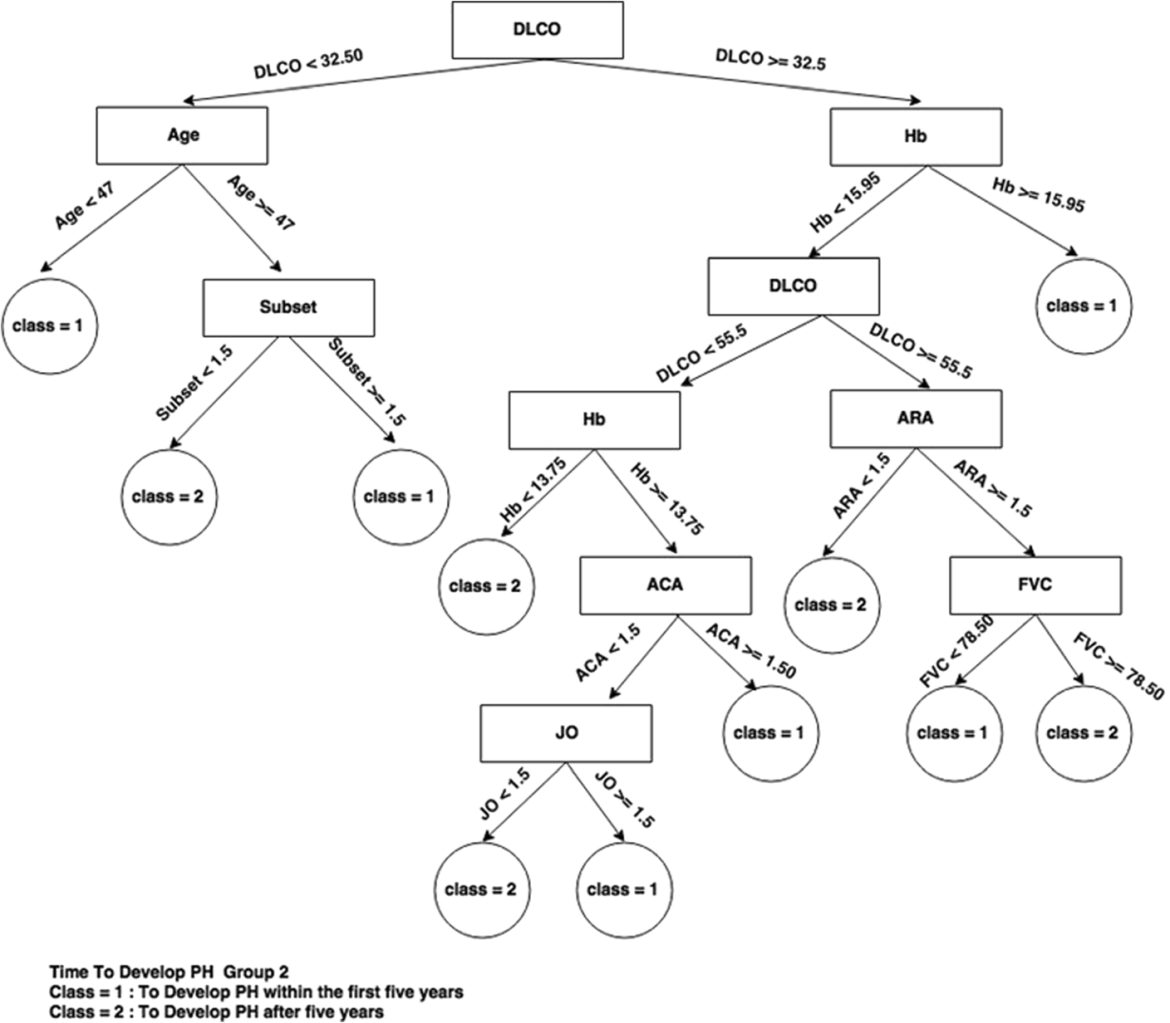
Consensus clustering decision tree for group 2 in SS dataset and time to develop pulmonary arterial hypertension class

**Fig. 6:**
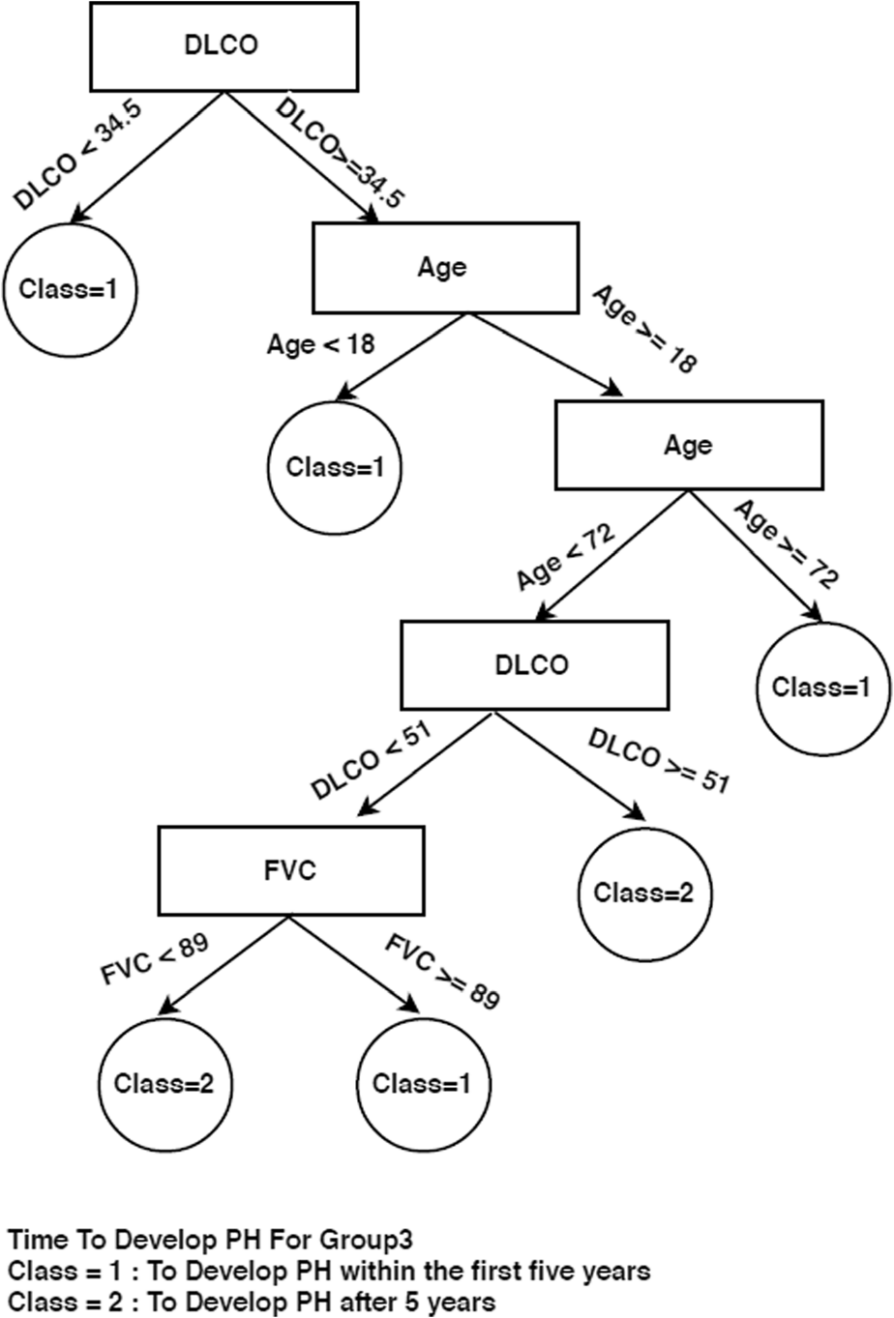
Consensus clustering decision tree for group 3 in SS Dataset and time to develop pulmonary arterial hypertension class

If we now look at the decision trees inferred from each consensus cluster found in SS dataset when time to develop pulmonary arterial hypertension class needs to be predicted (Figs. 4, 5, and 6), we can see that the trees are very different, indicating a different set of required criteria for each subset of patients that have been discovered. For example, group 1 is considerably smaller than group 2 and group3 and all trees involve different combinations of important variables. This highlights the importance of separating out these cohorts of patients when diagnosing. For instance, in group 3, knowing the DLCO, age, and FVC test result, has more of an impact on predicting time to develop pulmonary arterial hypertension whereas in group 1, knowing only the Hb, ACA, and others has more impact for predicting time to develop pulmonary arterial hypertension. Figure 5 is very simple decision tree that only rely on the Hb variable, so from the Hb attribute values for the first group, time to develop pulmonary arterial hypertension can be predicted.

If we now explore the difference between the attributes in each discovered consensus cluster, we see notable differences (Table 1). It looks like that Cr (which is the value indicating the measure of creatinine in that test) in group 2 is smaller than group 1 and group 3. When Cr is greater than 90, this means it is normal and this is what we have found in group 2 and group 3, but when it goes below this value, it is not normal. The reference range for the time period for Cr was 60–97 μmol/L. Interestingly, these features do not appear in the decision trees, perhaps because they have been separated already by the identification of the different subgroups. By identifying these different subgroups and exploring their characteristics, we can better understand how they differ and what focused tests may be more appropriate for different patients when making prognoses. By identifying the characteristics of each consensus cluster, we can identify the likelihood of patients belonging to any of these cohorts and apply more appropriate clinical tests as identified in the cohort-specific decision trees. This is essentially what the algorithm does when in the testing phase.

**Table 1:** Proportion/means values for SS attributes in CC (time to develop PAH)

	Group1	Group2	Group3
	Proportion		
Subset (without skin thickening)	56%	62%	55%
Subset (with skin thickening)	44%	38%	45%
Gender male	16%	16%	14%
Gender female	84%	84%	86%
	Proportion (patients have an event)		
ACA	**22%**	**28%**	**35%**
ATA	20%	20%	18%
ARA	**15%**	**1%**	**12%**
U3RNP	**4%**	**6%**	**0%**
NRNP	10%	6%	4%
PMSCL	4%	4%	6%
Th-RNP	0%	2%	0%
KU	1%	3%	0%
Jo1	2%	3%	0%
RO	4%	7%	8%
LA	1%	1%	6%
SM	0%	0%	1%
DSDNA	2%	1%	0%
ANA	18%	16%	12%
ANA NEG.	2%	4%	6%
	Means		
	Group 1	Group 2	Group 3
Hb	12.59	12.78	12.71
Cr	**97.06**	**84.53**	**93.46**
FVC	88.52	89.32	90.37
DLCO	65.58	63.38	65.48
Age	48.11	48.3	49.91

We now explore disease-free survival analysis: the Kaplan-Meier estimator, also known as product limit estimator, is a nonparametric statistic method used to estimate the survival function in reference to an event of interest, such as death or a disease complication [32]. The estimator is plotted over time to obtain the Kaplan-Meier curve, which is constituted by a series of horizontal steps of declining magnitude that, when a large sample is taken, approaches the true survival function for that population. The curve can be estimated easily if the patient is followed until death by computing the fraction surviving at each time, but in most cases, there are a number of patients that tend to drop out for different reasons. Nevertheless, the Kaplan-Meier analysis allows this information from both censored and uncensored observations to be considered, and the dependent variable is composed of two parts, the time to event and the event status, which records if the event of interest occurred or not. Censored data is where the event is only partially known because it has not happened yet—for example, in the SSc data, we may only know that a patient has not developed pulmonary arterial hypertension for *at least* X years at any point in time. The Kaplan-Meier curve is defined as the probability of surviving in a given length of time while considering time in many small intervals, taking into account only three weak hypotheses [33]. It is required to assume that the censored patients are characterized by the same survival prospects as those who continued to be followed, that the survival probabilities are the same for patients recruited early and late in the study, and finally that the event of interest happens at the specified time [32].

We carry out a survival analysis in order to determine how long a patient survives or how long from diagnosis before a patient develops a disease-associated internal organ complication, in relation to the discovered subgroups. By grouping subjects based on the nearest consensus clustering classification, we can then analyze if the discovered clusters are able to separate systemic sclerosis patients into subpopulations that show different symptoms and disease progression, for helping physicians to make better informed diagnosis and more focused interventions.

The following graph shows the percentage of patients survived from that organ complication on the *y*-axis, while on the *x*-axis the time to development of pulmonary arterial hypertension measured in months.

**Fig. 7:**
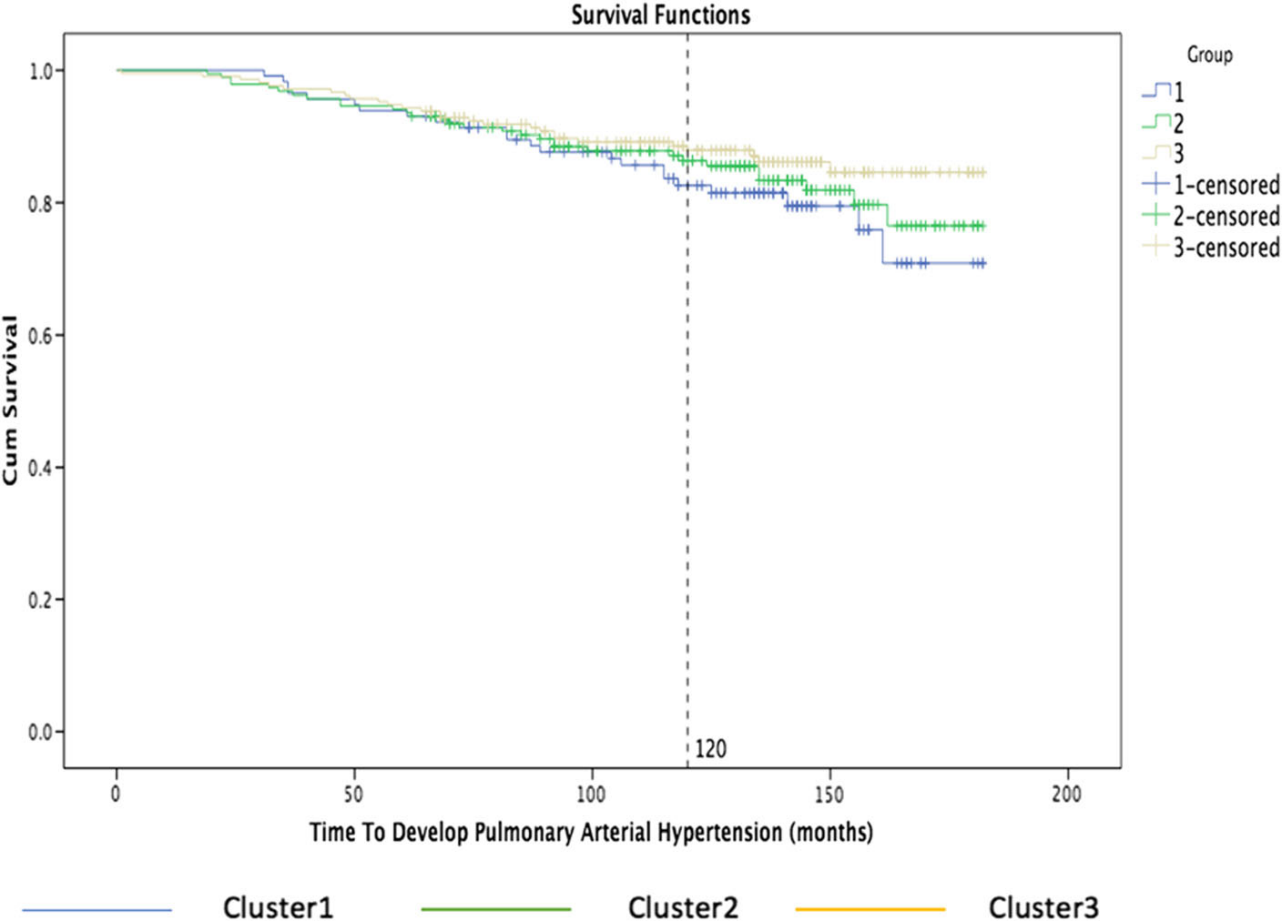
Kaplan-Meier curves by nearest consensus clustering on time to develop pulmonary arterial hypertension dataset. With time to develop pulmonary arterial hypertension in months on the *x*-axis and percentage of patients survived from that organ complication on the *y*-axis, the graph illustrates the survival curves obtained grouping patients based on nearest consensus clustering

Figure 7 shows the Kaplan-Meier curves for the three main clusters: cluster 1 is blue, cluster 2 is green, and cluster 3 is yellow. The graph shows clearly that 18% of the patients with the third group were affected of pulmonary hypertension after 120 months while about 10% of the patients were affected after 120 months in the first group.

### Systemic Sclerosis: Time to Death

**Fig. 8:**
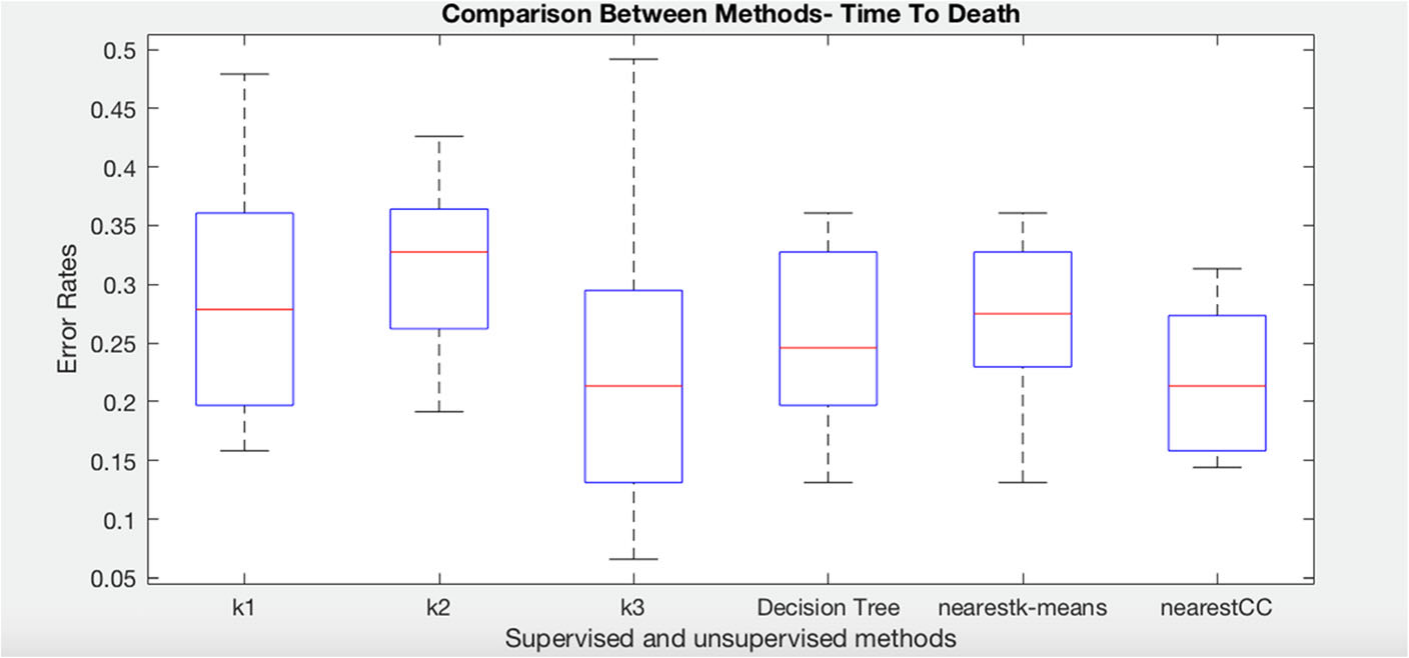
Comparison of K-means, decision tree, nearest K-means, and nearest CC for time to death class in systemic sclerosis dataset

We have repeated the same algorithms in order to predict time to death (T2RIP). The dataset was divided into three groups. Note that these groups are not the same as for the T2PAH experiments as the data selected will be different. The following boxplot (Fig. 8) shows that nearest consensus clustering classification performs better than nearest K-means although nearest K-means has less variation (*t* test *p* value = 0.041).

The consensus clustering decision trees which predict time to death class for the three groups of patients can be found in the Electronic Supplementary Material.

**Fig. 9:**
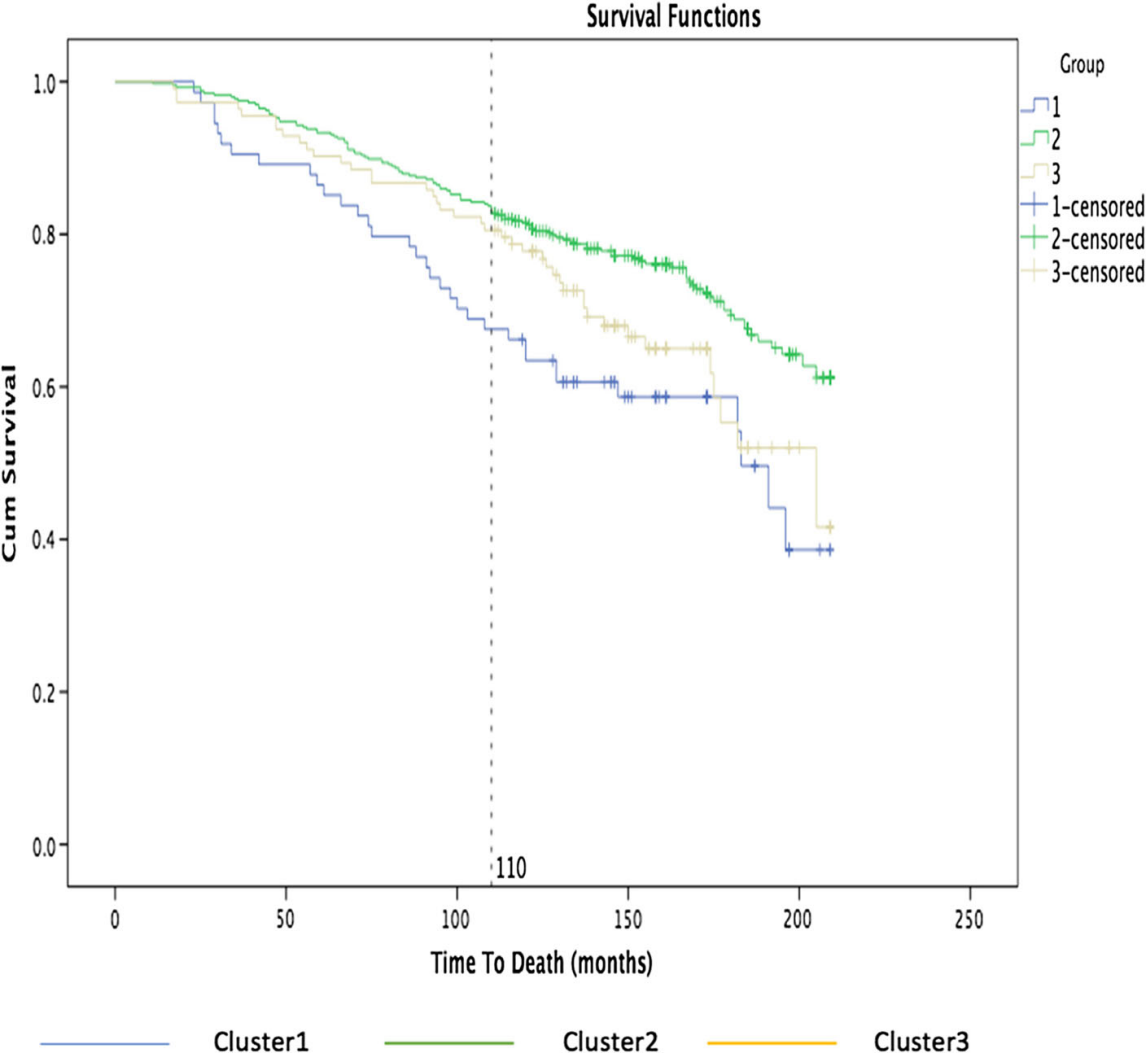
Kaplan-Meier curves by nearest consensus clustering on *time to death dataset*. With *time to death* in months on the *x*-axis and percentage of patients survived on the *y*-axis, the graph illustrates the survival curves obtained grouping patients based on nearest consensus clustering

Figure 9 shows that almost 35% of the patients from the first group died after 110 months while 15% of patients from groups 2 and 3 died.

Again, we see notable differences between the attributes in each discovered consensus (Table 2). It looks like that Cr (which is the value indicating the measure of creatinine in that test) in group 1 is smaller than group 1 and group 3. When Cr is greater than 90, this means it is normal and this is what we have found in group 2 and group 3, but when it goes below this value, it is not normal. The baseline that has been used in order to distinguish between the groups is whereas Cr is normal or not. Also, it is clear that DLCO is the smallest in the third group.

**Table 2:** Proportion/mean values for SS attributes in CC (time to death)

	Group1	Group2	Group3
	Proportion		
Subset (without skin thickening)	55%	56%	56%
Subset (with skin thickening)	45%	44%	44%
Gender male	12%	2%	18%
Gender female	88%	98%	82%
	Proportion (patients have an event)		
ACA	**24%**	**25%**	**25%**
ATA	23%	24%	22%
ARA	**13%**	**10%**	**10%**
U3RNP	**4%**	**6%**	**5%**
NRNP	7%	4%	8%
PMSCL	2%	7%	4%
Th-RNP	0%	1%	2%
KU	1%	1%	2%
Jo1	1%	1%	1%
RO	4%	6%	7%
LA	1%	2%	1%
SM	0%	0%	1%
DSDNA	1%	1%	1%
ANA	21%	5%	18%
ANA NEG.	1%	4%	4%
	Means		
	Group1	Group2	Group3
Hb	12.72	12.53	12.58
Cr	**87.41**	**93**	**96.28**
FVC	87.21	88.65	87.33
DLCO	66	64	62.56
Age	48	51	49

Other decision trees for time to death subgroups can be found in Electronic Supplementary Material.

In order to aid reproducibility, we now explore the freely available breast cancer dataset available from the UCI repository. K-means, decision tree, nearest K-means, and nearest CC classification were applied in order to predict whether the tumor was malignant or benign. The results can be found completely in Electronic Supplementary Material.

### Sensitivity Analysis

Specificity, sensitivity, precision, and recall have been used to evaluate the results. We have computed all of these measures for K-means, decision tree, nearest K-means, and nearest CC classification for time to develop pulmonary arterial hypertension and BC dataset results. Tables 3 and 4 show the results.

**Table 3:** Metrics measures results for three K-means groups, decision tree, nearest K-means, and nearest CC for time to develop pulmonary arterial hypertension class

	K1	K2	K3	DT	NKDT	NCCC
Sensitivity	0.5921	0.5345	0.5144	0.7233	0.7322	0.7544
Specificity	0.6921	0.6537	0.6745	0.8021	0.8021	0.8256
Precision	0.6211	0.5534	0.4688	0.7234	0.7133	0.8134
Recall	0.5921	0.5345	0.5144	0.7233	0.7322	0.7544

**Table 4:** Metrics measures results for three K-means groups, decision tree, nearest K-means, and nearest CC for BC dataset

	K1	K2	K3	DT	NKDT	NCCC
Sensitivity	0.7421	0.7178	0.8416	0.7822	0.7422	0.8311
Specificity	0.7832	0.7432	0.8643	0.8012	0.7721	0.8532
Precision	0.7934	0.7323	0.8711	0.8321	0.7895	0.8687
Recall	0.7421	0.7178	0.8416	0.7822	0.7422	0.8311

The above results show that nearest consensus clustering classification has improved the learning significantly as it looks like that nearest consensus clustering perform much better than K-means, decision tree, and nearest K-means.

### Impact of Different Number of Clusters (K)

**Fig. 10:**
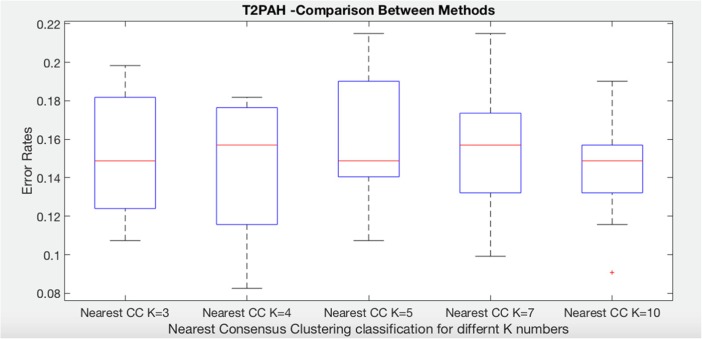
Comparison of nearest CC classification for time to develop pulmonary arterial hypertension class with different values of K

We briefly explore the effect of different values of K (K-means clustering method) on accuracy. We have run nearest consensus cluster classification to the systemic sclerosis data in order to predict time to develop pulmonary arterial hypertension and time to death five times for each class as each time K has different value. The following two plots show the results of these experiments as well as the result of each individual consensus cluster classification model on all of the test data (K = 3, K = 4, K = 5, K = 7, K = 10). Regarding time to develop pulmonary arterial hypertension, notice first that nearest consensus cluster classification for K = 3 (NCC3) and K = 4 (NCC4) classify the test data quite similar than the others and perform better than NCC5 and NCC7, while the NCC10 improves error and grown variation but it has noise (Fig. 10).

**Fig. 11:**
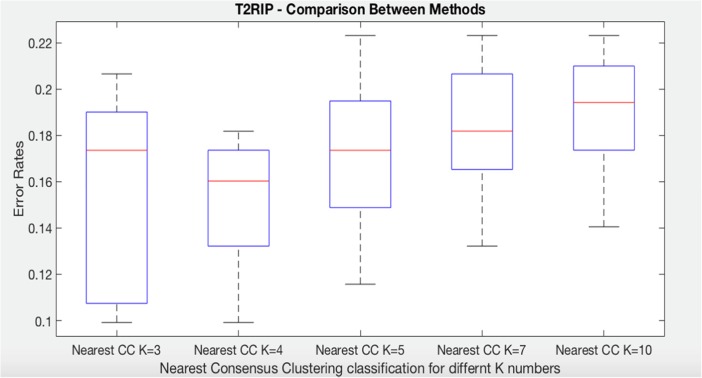
Comparison of nearest CC classification for time to death class with different values of K

In relation to time to death (T2RIP), notice first that nearest consensus cluster classification for K = 4 (NCC4) perform better and less variation than K = 3 (NCC3). Also, NCC4 classifies the test data better than NCC5, NCC7, and NCC10 (Fig. 11).

### Comparison to Other Clusterings/Classifiers

Finally, we briefly compare our new method with some other cluster/classifier combinations including support vector machine when it runs individually and when it merges with K-means in order to make sure if the proposed method performs better or not, and also, hierarchical clustering decision tree and PAM decision tree. Table 5 shows the results.

**Table 5:** Accuracy comparison between the proposed algorithm and others

	Time to death	Time to develop
Classifier	Accuracy	Accuracy
Decision tree	0.754	0.701
Nearest K-means	0.724	0.696
Nearest CC	0.781	0.722
SVM	0.721	0.689
SVM_K-means	0.752	0.711
Hierarchical clustering DT	0.713	0.725
PAMDT	0.749	0.731

## Conclusions and Future Work

In this paper, a set of algorithms were tested on systemic sclerosis dataset and breast cancer for simultaneously identifying subgroups of patients and diagnosing them based on these subgroups. The results illustrate issues with firstly ignoring the existence of subgroups of patients (with worse error rates) and secondly using standard clustering methods such as K-means (with higher variance in errors due to sample variance and method bias). The paper introduces a novel approach that exploits consensus clustering methods and single linkage distance metrics to deal with these issues. Our method, nearest consensus clustering classification integrates decision trees, consensus clustering, and single linkage metrics which has improved the classification and reduced the variance when tested on breast cancer data from the UCI repository and a dataset for systemic sclerosis from the Royal Free hospital in London. This new model can be used by clinics to cluster patients and discover key features in each group for classifying more confidently.

Future work will look at using other resampling methods, a further exploration of other linkage methods, and kappa measures to identify relationships between resampled cluster distances and the associated classification accuracies. We would also like to explore the prediction of other complications and how they interact using multiclass models.

## Electronic Supplementary Material

ESM 1 (DOCX 345 kb)
