# Supplementary material for: Nearest Consensus Clustering Classification to Identify Subclasses and Predict Disease
Source: J Healthc Inform Res. 2018 Jul 30;2(4):402–22. doi: 10.1007/s41666-018-0029-6 (PMC6245235; doi:10.1007/s41666-018-0029-6)
Supplement: Supplementary file 1 — (DOCX 345 kb) [file 41666_2018_29_MOESM1_ESM.docx]

**Supplementary Material for the Paper “Nearest Consensus Clustering Classification to Identify Subclasses and Predict Disease”**

Awad A Alyousef^1^, Svetlana Nihtyanova^2^, Chris Denton^2^, Pietro Bosoni^3^, Riccardo Bellazzi^3,^ Allan Tucker^1^

^1^ Dept Computer Science, Brunel University London, UK

^2^ UCL Royal Free Hospital, London, UK

^3^ University of Pavia, Italy
[awad.alsaidalyousef@brunel.ac.uk](mailto:awad.alsaidalyousef@brunel.ac.uk)

1. Summarry

This supplementary metrical contains additional consensus clustering decsion trees that have been resulted for Systemic sclerosis disease. It shows the decision trees for time to death class. Also, it contains the whole results for Breast Cancer that have been found when Nearest Consensus Clustering Classification and other algorithms were applied in order to predict whether the tumour was malignant or benign.

1. Systemic Sclerosis Time to Death

The following decision trees show the variations for each group of the patients when Time to death needs to be predicted.


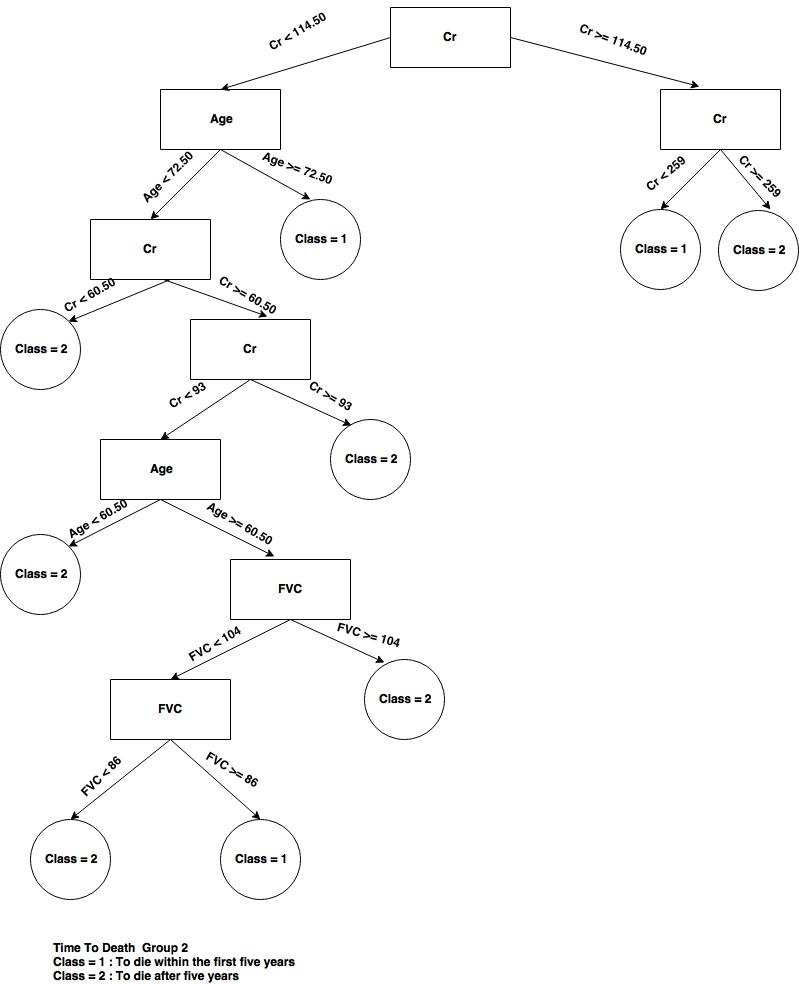


**Fig.A1**. Consensus Clustering Decision Tree for group 2 in SS Dataset and *Time to Death* class.


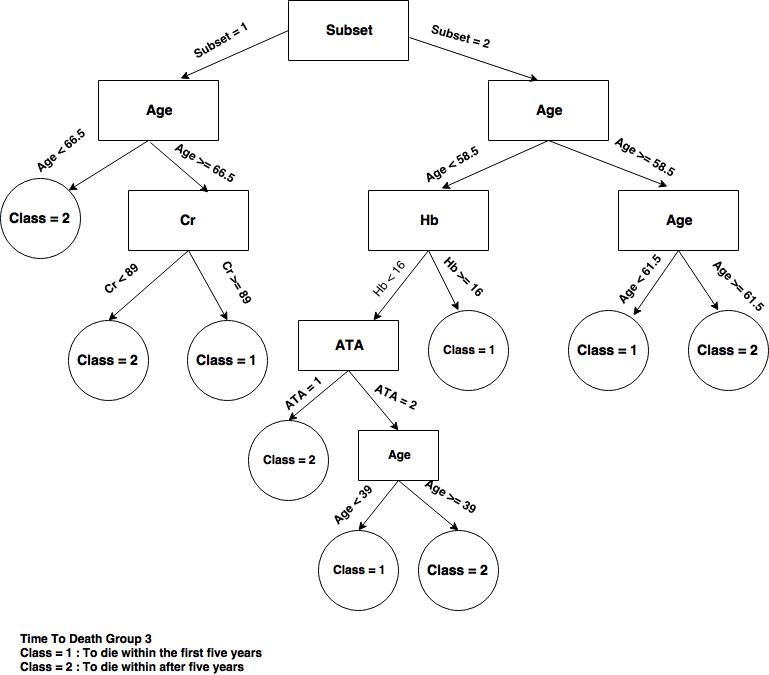


**Fig.A2**. Consensus Clustering Decision Tree for group 3 in SS Dataset and *Time to Death* class


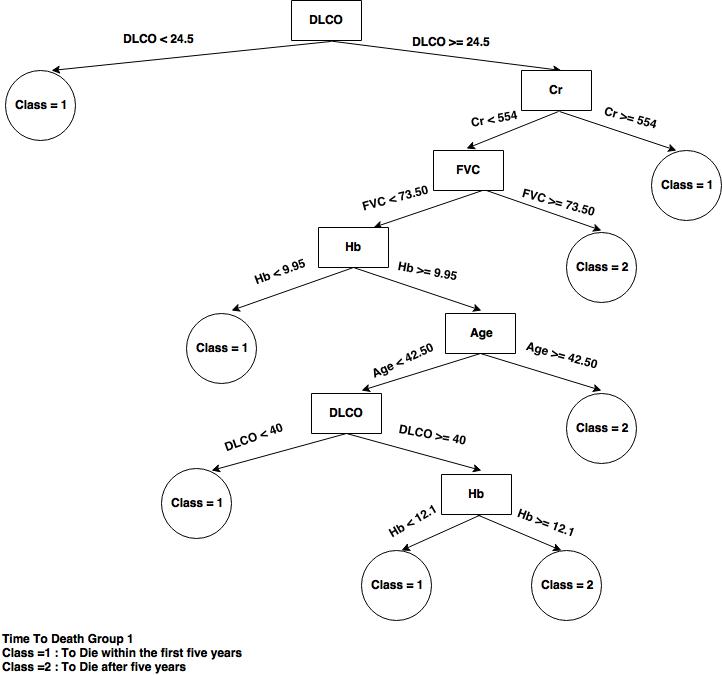


**Fig.A3**. Consensus Clustering Decision Tree for group 1 in SS Dataset and *Time to Death* class.

.

1. Breast Cancer: Tumour Type

In order to aid reproducibility, we now explore the freely available breast cancer dataset available from the UCI repository. K-means, Decision Tree, Nearest K-means and Nearest CC classification were applied in order to predict whether the tumour was malignant or benign. Again, the results in Fig. A4 shows that Nearest CC classification perform better than K-means and standard decision tree.


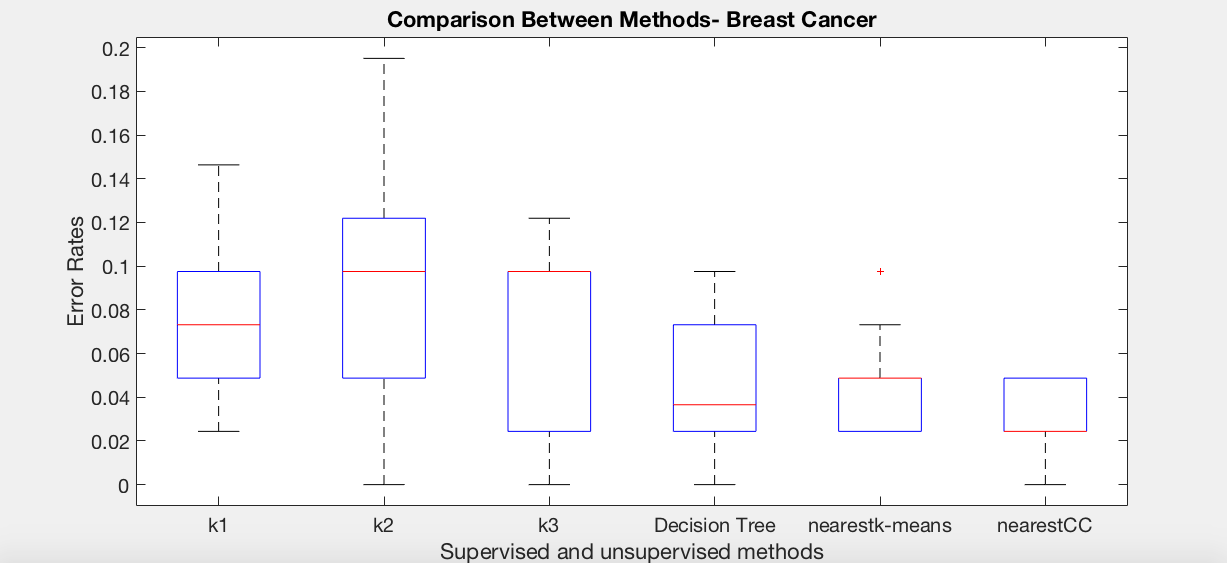


**Fig.A4.** Comparison of K-means, Decision Tree, Nearest K-means, and Nearest CC Classification for BC dataset

Fig. A5 shows the decision trees that predicts the Breast Cancer within group 1. BC whether it is benign or malignant can be predicted easily for all patients who are within group 1 by knowing Cell shape and Cell size. Also, it shows that Thickness is necessary for group 2 patients in order to predict BC.


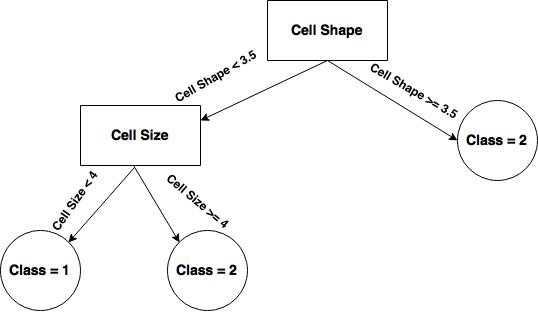


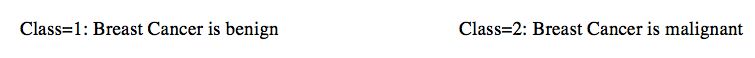


**Fig.A5**. Consensus Clustering DT for Breast Cancer prediction Group1


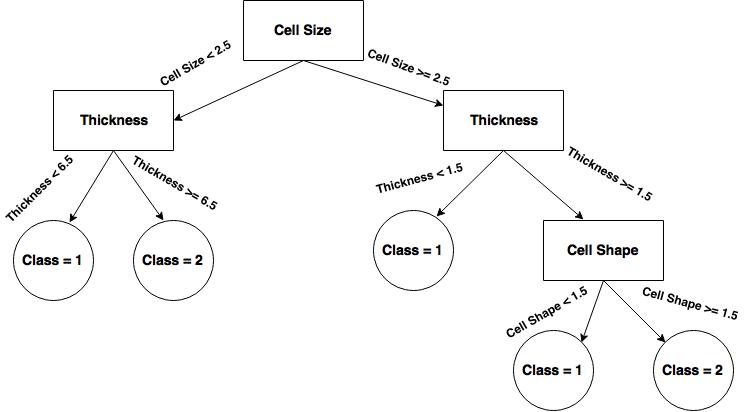


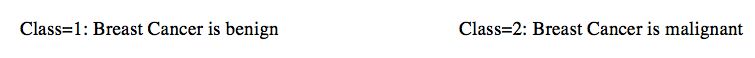


**Fig.A6**. Consensus Clustering DT for Breast Cancer prediction Group2


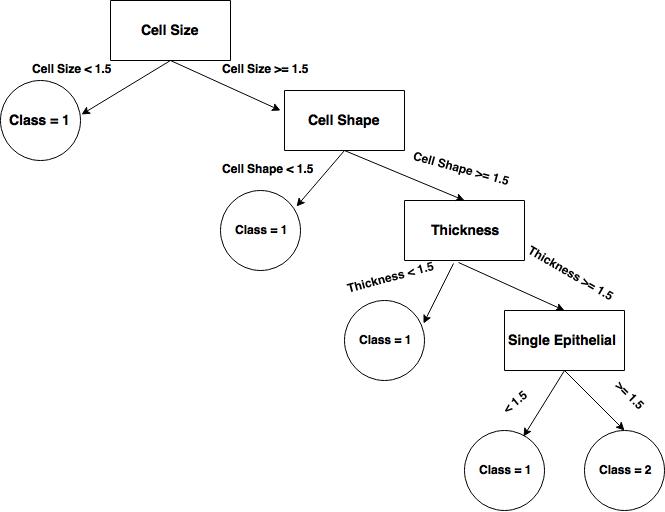


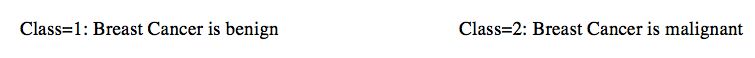


**Fig.A7.** Consensus Clustering DT for Breast Cancer prediction Group3

Table A1 shows the variation of attributes in each group. It looks like that Thickness values in Group1 is greater than its values in Group 2 and Group 3. Also, cell size values in Group 3 are smaller than its values in Group1 and group 2.

The same above experiments have been applied to predict Breast Cancer but the dataset has been divided into 4 groups. Fig.A8 shows the comparison between all algorithms. It shows how well Nearest Consensus Clustering Classification performs (note that here the t-test p-value is 0.050 so borderline significantly different from nearest K-Means but with a lower mean).


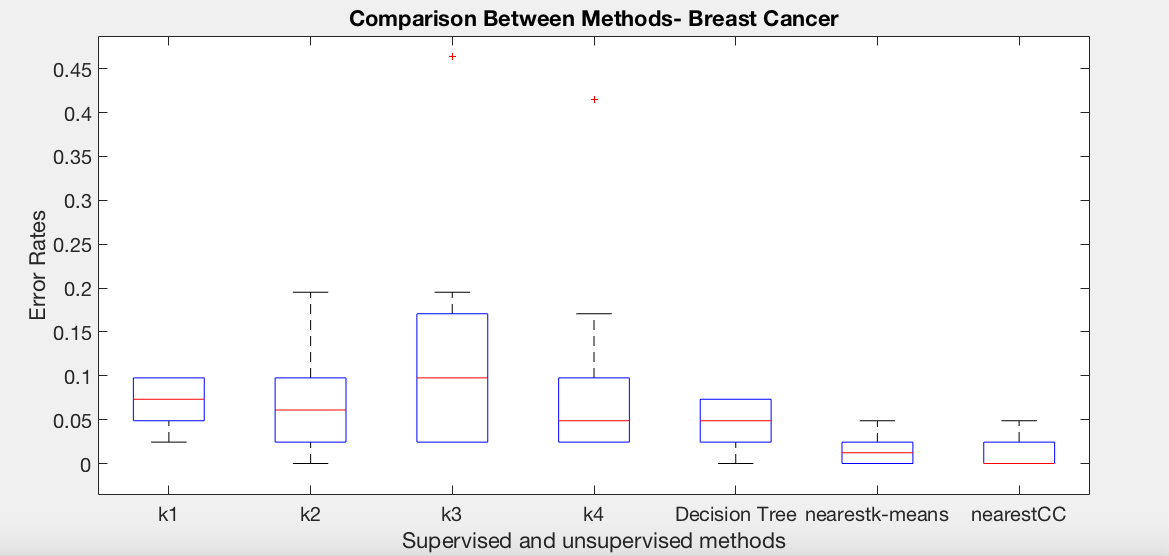


**Fig.A8.** Comparison of K-means, Decision Tree, Nearest K-means, and Nearest CC Classification for BC dataset (four groups)

**Table A1**. Means for BC attributes in Consensus Clustering
